# Responding to a pandemic with Simulation Based Education (SBE)? Sharing lessons learned from Sussex Partnership Foundation Trust (SPFT)

**DOI:** 10.1192/bjo.2021.415

**Published:** 2021-06-18

**Authors:** Craig McEwan, Richard Kerslake, Michael Hobkirk

**Affiliations:** Sussex Partnership NHS Foundation Trust (SPFT)

## Abstract

**Aims:**

In March 2020 SPFT was preparing for the first wave of the COVID-19 pandemic. Senior medical leadership supported the rapid development and delivery of SBE workshop for assessment and management of physically unwell patients in a psychiatric setting in the context of COVID-19. The training was delivered to 102 psychiatrists across 10 sessions over 4 weeks.

A learning review was completed to identify lessons learned from the delivery of this SBE workshop.

**Method:**

The intervention was reviewed using open-space feedback from attendees, interviews with facilitators and medical leadership, and SWOT (Strengths, Weaknesses, Opportunities, Threats) analysis.

**Result:**

Overall, the simulation project met its pre-determined objectives of increasing confidence and competence in the medical workforce in the context of COVID-19 and physical health. Development and delivery of the workshop was rapid, with request to delivery taking 4 days.

A summary of the key lessons include:

An existing simulation faculty within the trust was essential, allowing for rapid identification of key stakeholders and those able to deliver the project.

A “direct-line” relationship to senior leadership enabled the project to be dynamic and responsive to changing demands as COVID-19 guidelines and objectives evolved.

Redeploying higher trainees with SBE experience to develop the project as a focussed team allowed for rapid delivery which was resource-effective.

The workforce found reassurance from understanding what was not expected of them, as much as what was. For example, making clear that Arterial Blood Gases would not be introduced to the psychiatric setting.

There is an ongoing learning need for physical health training through SBE in non-covid scenarios.

SBE can be an effective intervention for a range of medical grades and covering a large geographical area.

There are opportunities for developing multi-disciplinary training on physical health in psychiatry.

**Conclusion:**

We have outlined some of the key learning outcomes from a successfully implemented SBE project during the first COVID-19 wave in spring 2020. The project has cemented the role of the relatively new simulation faculty within the trust and highlighted the effectiveness of close collaboration between leadership and a small, dedicated group of facilitators. The project has continued to be used for training new staff members and the resources have been widely shared, used by other NHS trusts and also internationally.

